# Root Morphological Traits of Seedlings Are Predictors of Seed Yield and Quality in Winter Oilseed Rape Hybrid Cultivars

**DOI:** 10.3389/fpls.2020.568009

**Published:** 2020-10-15

**Authors:** Julien Louvieaux, Martin Spanoghe, Christian Hermans

**Affiliations:** ^1^Crop Production and Biostimulation Laboratory, Interfacultary School of Bioengineers, Université Libre de Bruxelles, Brussels, Belgium; ^2^Laboratory of Applied Plant Ecophysiology, Haute Ecole Provinciale de Hainaut Condorcet, Centre pour l’Agronomie et l’Agro-Industrie de la Province de Hainaut, Ath, Belgium; ^3^Laboratory of Biotechnology and Applied Biology, Haute Ecole Provinciale de Hainaut Condorcet, Mons, Belgium

**Keywords:** *Brassica napus*, field performance, genetic diversity, hydroponics, root morphology

## Abstract

The root system is responsible for soil resources acquisition. Hence, optimizing crop root characteristics has considerable implications for agricultural production. This study evaluated a panel of twenty-eight European modern cultivars of oilseed rape (*Brassica napus* L.) cultivated in laboratory and field environments. Root morphology was screened using a high-throughput hydroponic growth system with two divergent nitrogen supplies. The panel showed an important diversity for biomass production and root morphological traits. Differences in root and shoot dry biomasses and lateral root length were mainly explained by the genotype, and differences in primary root length by nitrogen nutrition. The cultivars were tested in a pluriannual field trial. The field variation for yield and seed quality traits attributed to the genotype was more important than the year or the genotype × year interaction effects. The total root length measured at the seedling stage could predict the proportion of nitrogen taken up from the field and reallocated to seed organs, a component of the nitrogen use efficiency. The genetic interrelationship between cultivars, established with simple sequence repeat markers, indicated a very narrow genetic base. Positive correlations were found between the genetic distance measures, root morphological traits during nitrogen depletion and yield components. This study illustrates a root phenotyping screen in the laboratory with a proof of concept evaluation in the field. The results could assist future genetic improvements in oilseed rape for desirable root characteristics to reduce nutrient losses in the environment.

## Introduction

Nitrogen (N) is the nutrient required in the greatest amount for plant growth and the most determining one for crop yield (Lasisi et al., 2018; Qin et al., 2019). From the soil, plants can absorb mainly inorganic N forms (nitrate and ammonium) and N-containing organic compounds (amino acids and peptides) (Näsholm et al., 2009; Tegeder and Rentsch, 2010). In agricultural soils, inorganic N forms are prevailing (Jämtgård et al., 2010; Lonhienne et al., 2014). Since 1960, the N fertilizer consumption has increased worldwide nearly ten times (IFASTAT, 2017), while the global crop demand is expected to double by 2050 (Tilman et al., 2011). Sustainable agriculture faces the challenge to produce more food while reducing the negative environmental impact of N fertilization. Cultural practices (e.g., precise fertilization, split doses or matching fertilizer forms) and crop genetic improvement are different levers for better use of N sources (Kant et al., 2011; Han et al., 2015). Besides, understanding the mechanisms of crop adaptation to N availability is crucial for improving Nitrogen Use Efficiency (NUE). The NUE has two main components: the Nitrogen Uptake Efficiency (NUpE) and the Nitrogen Utilization Efficiency (NUtE). These are, respectively, describing the capacity to acquire N from the soil and to utilize the absorbed N for producing harvestable organs (reviewed in Han et al., 2015). The latter one can be divided into the Nitrogen Assimilation Efficiency (NAE) and the Nitrogen Remobilization Efficiency (NRE).

Plant roots fulfill important functions as they not only provide anchorage but also forage soil for water and nutrients. The root system architecture defines the spatial distribution of roots in the soil expressing the ability of the plant to acquire soil resources and is plastic in response to N availability (Smith and De, 2012). In the model species *Arabidopsis thaliana*, a dual effect of nitrate on lateral root (LR) development is described by: (i) a systemic inhibition of uniformly elevated nitrate concentrations occurring on LR elongation at the post-emergence developmental stage and (ii) a localized stimulation of nitrate-rich patches triggering LR elongation of N-deficient plants, known as the foraging capacity (Zhang and Forde, 1998; Zhang et al., 1999; Ruffel et al., 2011). The repression of root development during important N input results in a suboptimal soil volume exploration (López-Bucio et al., 2003; Gruber et al., 2013; Qin et al., 2019). Modern crop breeding is exploiting the natural variation of root morphology to enhance crop productivity, nutrient and water use efficiencies, and to reduce N fertilizer input (Garnett et al., 2009; White et al., 2013; He et al., 2019).

Oilseed rape (*Brassica napus* L.) is the second most important oilseed crop worldwide after soybean, and the first one in Europe (Stahl et al., 2017). That crop requires an important N input and has poor NUE, with a low seed production per N unit applied (Rathke et al., 2005; Sylvester-Bradley and Kindred, 2009; Ulas et al., 2013). The recent domestication of oilseed rape has suffered from several genetic diversity bottlenecks, due to the selection of modern varieties with low concentrations of erucic acid and glucosinolate (Bouchet et al., 2016; Stahl et al., 2017; Hatzig et al., 2018). Surveys showed that crop yield gain has negatively impacted on root system size (Aziz et al., 2017; Pérez-Jaramillo et al., 2017; Bektas and Waines, 2018) and the green revolution unintentionally selected towards poor root morphological features (Voss-Fels et al., 2017). Some reports indicate that N-efficient oilseed rape cultivars are characterized by an important root density during the vegetative growth stage (Ulas et al., 2012). In addition, the genotypic variation of winter oilseed rape for NRE is less substantial than that of NUpE (Ulas et al., 2013), encouraging exploration of the second one. Only few reports associated the root phenome to the field performance of oilseed rape (White et al., 2013; Thomas et al., 2016b; Louvieaux et al., 2020).

This study was conducted with a diversity panel of 28 modern winter oilseed rape cultivars cultivated in laboratory and field environments. Our approach was (i) to explore the natural variation for root morphology at the seedling stage with two contrasting N supplies, and to identify root traits accounting for most of the variation (ii) to examine yield components in a pluriannual field trial (iii) to compare seedling with adult plant traits and to assess the predictiveness of laboratory observations for field performance and (iv) to evaluate relationships between genetic distances based on molecular markers and distances computed from phenotypic data.

## Materials and Methods

### Plant Material

A panel of twenty-eight cultivars of winter oilseed rape (*Brassica napus* L.), registered in the European catalogs of plant species and varieties for less than 10 years, was assembled (Supplementary Table 1). Seeds were obtained from Terres Inovia (France). The cultivars were part of the trial network for the period covering 2015 to 2019 for post-registration evaluation of winter oilseed rape cultivars in northern France. The diversity panel reflects the trend of commercial oilseed rape varieties, with a prevalence of hybrids over the past two decades (Stahl et al., 2017). These genotypes undeniably outperform older ones for seed yield (Kessel et al., 2012; Koscielny and Gulden, 2012; Koeslin-Findeklee et al., 2014; Stahl et al., 2017). The mean seed weight at sowing (MSW) was determined by weighing three subsamples of 500 seeds of each cultivar.

### Laboratory Culture

A pouch and wick hydroponic system was used for phenotyping root morphology of seedlings (Thomas et al., 2016b; Louvieaux et al., 2018). The seeds were placed on a blue germination paper (grade 194, Ahlstrom-Munksjö, Bärenstein, Germany) soaked in distilled water and then, stratified for 1 week at 4°C. Seeds were then transferred to a culture chamber (Growbank XXL2, CLF Plant Climatics, Wertingen, Germany), where the temperature was 21°C, the light period 16 h (150 μmol photons m^–2^ s^–1^)/8 h darkness and the relative humidity 70%. Two days after germination, six seedlings of uniform size were placed onto one blue paper sheet (20 cm × 30 cm), covered with a black microperforated rigid plate (Biplex^®^, IPB, Waregem, Belgium) to overshadow the root organs. The mounts (two per genotype, corresponding to twelve seedlings) were placed in containers filled with 10 L of nutrient solution. After 4 days, root and shoot organs were separated, and the root systems scanned at 300 dpi (HP Photosmart C4100). Images were analyzed with the RootNav software (Pound et al., 2013) to extract root morphological traits (Table 1 and Supplementary Figure 1). Eventually, the dry weight of root and shoot organs was measured after 48 h at 70°C. The composition of the nutrient solution was adapted from Hermans et al. (2010). The nitrate concentration varied: the N− solution contained 0.2 mM nitrate [0.1 mM Ca(NO_3_)_2_ + 2.4 mM CaCl] and the N+ solution 5.0 mM nitrate [2.5 mM Ca(NO_3_)_2_].

**TABLE 1:** Definition of biomass production and root morphological traits measured in hydroponically grown plants.

Abbreviation	Description
R	Root dry biomass (mg)
S	Shoot dry biomass (mg)
R+S	Total dry biomass (mg)
R:S	Root to shoot biomass ratio
L_PR_	Length of primary root = L_Z2_+L_Z3_+L_Z4_ (cm)
L_Z2_	Length of primary root zone 2, defined between the first and last lateral roots (cm)
L_Z3_	Length of primary root zone 3, delimited between the hypocotyl junction and the first lateral root (cm)
L_Z4_	Length of primary root zone 4, delimited between the last lateral root and the primary root tip (cm)
N_LR_	Number of lateral roots >1 mm
ΣL_LR_	Sum of lateral root lengths (cm)
D_LR_-Z1	Density of lateral roots in zone 1 = N_LR_/L_PR_ (cm^–1^)
D_LR_-Z2	Density of lateral roots in zone 2 = (N_LR_−1)/L_Z2_ (cm^–1^)
TRL	Total root length = L_PR_+ΣL_LR_ (cm)
ML_LR_	Mean length of lateral roots = ΣL_LR_/N_LR_ (cm)
SRL	Specific root length = (L_PR_+ΣL_LR_)/R (cm mg^–1^)
MSW	Mean seed weight at sowing (mg)

*Illustration of the different root zones is shown in Supplementary Figure 1.*

### Field Culture

The field trials were conducted over four growing seasons (2015–2019) at the CARAH experimental farm in Ath, Belgium (50°36′48.089″ N, 3°45′58.186″ E). Annual rainfall is typically 863 mm, spread evenly over the year, and annual average temperature is 10.4°C (reference period 1980–2010). The four-year-period was characterized by less important rainfall (ranging from 362 mm in 2018 to 533 mm in 2016) and higher temperature (ranging from 10.8°C in 2016 to 11.6°C in 2018) than average. The silt-loam soil is classified as Luvisol with a favorable drainage.

Preceding crop was winter wheat. The sowing density was 60 seeds m^–2^. The culture received growth regulator and was protected against weeds, pests and diseases, as required. Each year, microplots of 1.5 m × 12 m in size were sown following a randomized complete block design with four replicates. Sowing dates were between August 28^th^ and September 8^th^ over the four-year-period. The field conditions were not N-limiting. Plots were fertilized with ammonium nitrate after winter, at vegetation stage BBCH 31–32. The fertilizer amount was adjusted on a yearly basis (ranging from 172 to 198 kg N ha^–1^ in 2016 and 2017, respectively), according to N absorbed in plant aerial biomass and mineral N in soil samples (0–90 cm profile) after winter, and using the predictive balance sheet method commonly used for the main arable crops (Makowski et al., 2005).

Cultivars were harvested at the same time, when the mean seed humidity (H) of the control varieties was less than 10% (between July 15th–30th over the 4-year period). To avoid side-effects, only the central parts of the microplots (1.5 m × 9 m) were harvested with a combine harvester (Wintersteiger Delta, Ried im Innkreis, Austria). Seed yield (SY) and seed quality traits are listed in Table 2. Subsamples of seeds from each replicate were analyzed for humidity at harvest (H), oil (OilConc), protein (ProtConc), and glucosinolate (GLS) concentrations by near infra-red spectroscopy (XDS NIR Analyzer, Foss, Hilleroed, Denmark). Specific weight of seeds (SW) was measured using a grain analyzer (GAC 2100, Dickey-John, Auburn, United States). A seed counter (Numigral, Chopin Technologies, Villeneuve-la-Garenne, France) was used to determine the thousand seed weight (TSW). Flowering earliness of cultivars (FLO) was visually estimated by the percentage of opened flowers on main inflorescences when control cultivars reached BBCH 65 (between April 10th–20th over the 4-year period). Within the same time window, the chlorophyll index (CHL), flavonol index (FLAV), anthocyanin index (ANTH) and the nitrogen balance index (NBI) were measured with a Dualex^®^ Scientific + leafclip (Force-A, Orsay, France), based on pigment fluorescence (Padilla et al., 2018; Louvieaux et al., 2020). Within each microplot, measurements were conducted on the young mature leaves of five individuals. For adjusting the year effect, a data matrix was computed by normalizing field traits to the mean of three control genotypes (Supplementary Table 1). These cultivars were selected among the most marketed and having a wide range of earliness at harvest, as recommended by the French Permanent Technical Committee for Plant Breeding (CTPS) and following protocol for official examination of Value for Cultivation and Use (VCU) of agricultural crops (Animal and Plant Health Agency, United Kingdom, 2016; CTPS, 2017).

**TABLE 2:** Definition of field traits.

Abbreviation	Description
***Seed yield and quality traits***	
SY	Seed yield corrected to a standard water content of 9% (t ha^–1^)
H	Humidity at harvest (%)
SW	Specific weight of seeds (kg hl^–1^)
TSW	Thousand seed weight with moisture adjusted to 9% (g)
OilConc	Oil concentration in dry seeds (%)
ProtConc	Protein concentration in dry seeds (%)
NConc	Nitrogen concentration in dry seeds = ProtConc/6.25 (%)
GLS	Glucosinolate concentration in dry seeds (μmol g^–1^)
OilY	Oil yield = OilConc × dry SY (t ha^–1^)
ProteinY	Protein yield = ProtConc × dry SY (t ha^–1^)
SNU	Seed nitrogen uptake = NConc × dry SY (kg ha^–1^)
FLO	Earliness of flowering (%)
***Optical indices measured at flowering***	
CHL	Chlorophyll index
FLAV	Flavonol index
ANTH	Anthocyanin index
NBI	Nitrogen balance index

### Genetic Survey

Genomic DNA was extracted from the cotyledons of two individuals per genotype, germinated in greenhouse conditions, using the DNeasy Plant Mini Kit following manufacturer’s protocol (Qiagen, Venlo, The Netherlands). The DNA samples were quantified with the ND-3300 NanoDrop spectrofluorometer (Thermo Fisher Scientific, Waltham, MA, United States).

Seventeen simple sequence repeat (SSR) markers were selected from the literature followed by in-depth internal testing based on the following criteria: (i) homogeneous repartition on the chromosomes; (ii) optimal amplification and resolution; (iii) capability to detect high rates of polymorphism; (iv) adequacy of observed fragment sizes with those reported in selected literature (Lowe et al., 2004; Cheng et al., 2009; Kim et al., 2009; Xu et al., 2010; Li et al., 2013; AAFC Consortium, 2016); and (v) suitability to be used in a multiplexed PCR reaction, according to the step-by-step protocol by Henegariu et al. (1997). These were amplified in two multiplex PCR sets of nine and eight SSR markers, respectively. The forward primer 5’ of each pair was labeled with a fluorescent dye (6-FAM, VIC, NED, or PET dyes) (Table 3). The PCR amplification was performed using the Kapa2G HotStart Multilocus Amplification Kit (Kapa Biosystems, Boston, United States) in 25 μl volumes containing 1.5× Kapa2G HotStart PCR Buffer, 0.2 mM dNTP, 0.1–0.2 μM of each primer (Thermo Fisher Scientific, Waltham, MA, United States), 1 unit of KAPA2G Fast HotStart DNA Polymerase and 15 ng of DNA template. All amplifications were done in the same conditions with the SimpliAmp Thermal Cycler (Applied Biosystems, Waltham, MA, United States). The PCR cycling parameters were as follows: initial denaturing step of 2 min at 95°C, 30 cycles of 15 s at 95°C, 30 s at 55°C (Ta), 12 s at 72°C, followed by a hold step of 2 min at 72°C for final extension. After amplification, 0.7 μl of PCR products for each multiplex PCR reaction were transferred into 14.3 μl HiDi (Applied Biosystems) containing 2% of GeneScan^TM^ 500 LIZ^TM^ dye Size Standard (Applied Biosystems), then denatured at 95°C for 3 min and quenched on ice. The denatured samples were run on the SeqStudio^TM^ Genetic Analyzer System (Applied Biosystems), following standard run module parameters. Estimations of PCR product lengths were determined using GeneMapper^TM^ Software 6.0 (Applied Biosystems).

**TABLE 3:** Genetic diversity information of the 17 SSR markers used for genotyping of 28 winter oilseed rape cultivars.

Marker Name	Multiplex set	Dye	Linkage group	Allele size range (bp)	No. of alleles	Ho	PIC
BrGMS4028^a^	1	VIC	A01	162–178	2	0.07	0.12
BrGMS0667^a^	1	PET	A02	173	1	0.00	0.00
sN2025^b^	1	6-FAM	A04	128–136	2	0.61	0.37
BrGMS0070^a^	1	NED	A05	188–227	7	0.82	0.76
BrGMS3750^a^	1	6-FAM	A06	209–214	2	0.29	0.28
BrGMS3837^a^	1	NED	A07	299	1	0.00	0.00
BnGMS0281^c^	1	PET	A09	277–295	6	0.61	0.57
BrGMS0086^a^	1	6-FAM	A10	298–314	3	0.43	0.34
BnGMS027^c^	1	VIC	C01	304–312	3	0.25	0.21
cnu_m250a^d^	2	NED	A03	203–264	5	0.54	0.48
Na14-G02^*e*^	2	6-FAM	A03	183–195	3	0.29	0.23
BrGMS0742^a^	2	NED	A08	131–139	3	0.39	0.34
BnGMS0347^*f*^	2	6-FAM	C04	272–278	4	0.57	0.47
Na12D10^*e*^	2	PET	C05	173	1	0.00	0.00
BnGMS0353^c^	2	PET	C06	286–302	4	0.14	0.13
Na12F03^*e*^	2	VIC	C07	305–315	5	0.61	0.56
Ol12D05^*e*^	2	VIC	C08	127	1	0.00	0.00
				**Average:**	**3.12**	**0.33**	**0.29**
				**Total:**	**53**		

*Ho, observed heterozygosity; PIC, polymorphism information content. Detailed marker information is available in: ^a^Xu et al., 2010; ^b^AAFC Consortium, 2016; ^c^Cheng et al., 2009; ^d^Kim et al., 2009; ^e^Lowe et al., 2004; ^f^Li et al., 2013.*

Polymorphism Information Content (PIC) was calculated for each marker as PIC = 1−Σ*Pi*^2^, where *Pi* is the frequency of the *i*th allele detected in the subset, according to the Nei’s statistic (Nei, 1973). Allelic frequency, observed heterozygosity, and PIC were computed using Cervus software v 3.0 (Kalinowski et al., 2007). Genotyping data was treated based on a hierarchical clustering analysis with the unweighted Neighbor Joining (NJ) methodology using the program DARwin 6.0 (Perrier and Jacquemoud-Collet, 2006) from a dissimilarity matrix beforehand computed by pair-wise comparisons based on simple matching of allelic data.

### Statistical Analysis

A two-way analysis of variance (ANOVA) was used to isolate the genotypic effect from the environment/nutrition effect in the laboratory experiment and from the year effect in field trials, as well as their interaction and residual effects. Furthermore, a principal component analysis (PCA) with laboratory and field traits was performed for capturing traits influencing the most the observed variability. Both ANOVA and PCA were executed using R software (R Core Team, 2014) with FactoMinerR (Lê et al., 2008), factoextra (Kassambara and Mundt, 2017), and corrplot (Wei and Simko, 2017) packages. Assumptions for ANOVA were verified with a D’Agostino-Pearson normality test. Correlations between traits were established with Pearson’s correlation method on R software, at significant level α = 0.05. Correlation plots were drawn with the corrplot package.

The Mantel test was used to investigate the relations between genetic dissimilarities matrix, computed from genotyping analysis, and trait dissimilarities matrices calculated as Euclidean distances from laboratory and field assays. Data were computed using ade4 package on R software at significance level α = 0.05 (parameter: 9999 permutations) (Chessel et al., 2004; Dray and Dufour, 2007; Dray et al., 2007; Bougeard and Dray, 2018).

## Results

### Laboratory Assays

#### Influence of the Nitrate Supply on Biomass Production and Root Morphology in a Laboratory Environment

**FIGURE 1:**
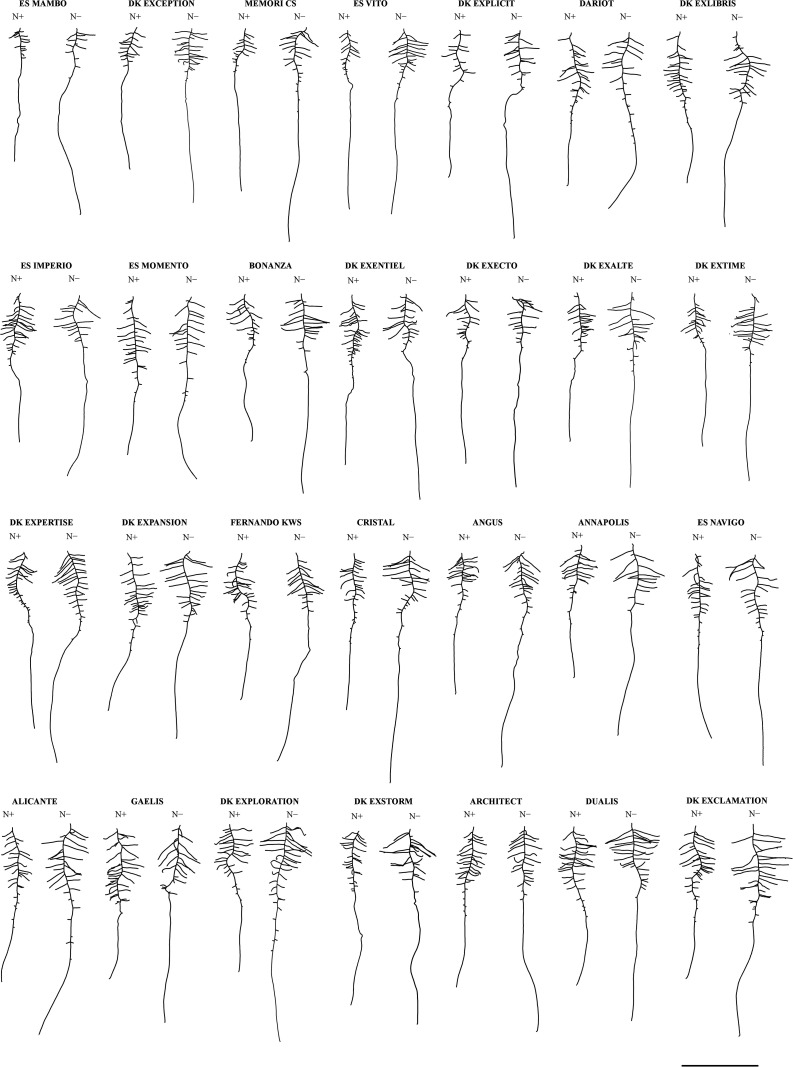
Representative root morphologies of 28 winter oilseed rape cultivars grown in hydroponics. Seedlings grew in the hydroponic pouch-and-wick system with 0.2 mM (N–) or 5.0 mM (N+) nitrate supplies (12 seedlings observed). Representative items for each cultivar-N concentration pair were selected from individuals being closest to the median value for both the primary root length (LPR) and the total root length (TRL). Cultivars are ordered by increasing total root length (TRL) measured at N–. Scale bar = 5 cm.

**FIGURE 2:**
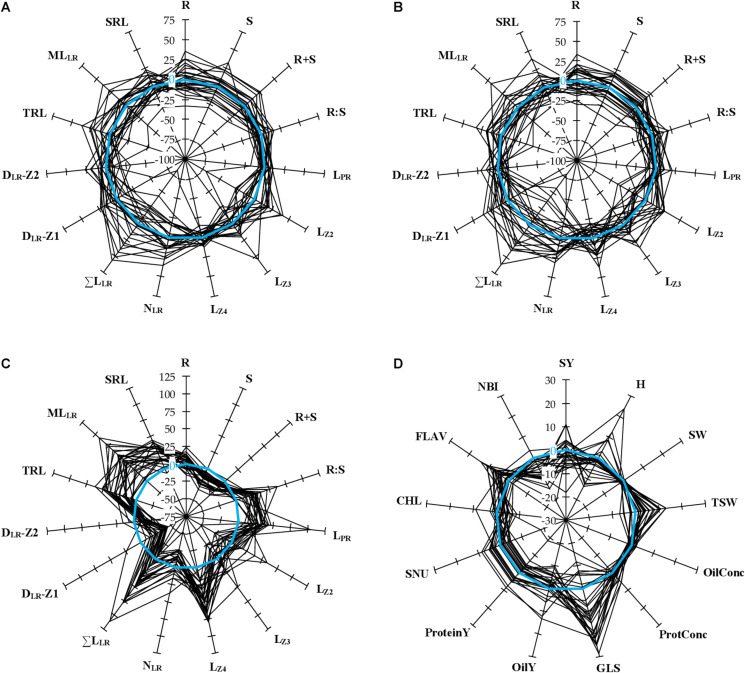
Relative variation of phenotypic traits measured in 28 winter oilseed rape cultivars. **(A,B)** The spider plots show the percentage variation of biomass production and root morphological traits for every cultivar, normalized by the mean value of the panel, measured during N– **(A)** or N+ **(B)** conditions. Zero percent (blue circle) indicates no difference compared to the mean value of the panel in one condition. **(C)** The spider plot shows the percentage variation of biomass production and root morphological traits for every cultivar grown under N– conditions, normalized by the value observed under N+ conditions. This defines the responsiveness of one trait to N depletion. Zero percent indicates no difference compared to N+ conditions. **(D)** The spider plot shows the percentage variation of field traits for every cultivar, normalized to the mean value of three reference cultivars (DK Exception, DK Expansion, and DK Exstorm) over four growing seasons. Zero percent indicates no difference compared to the reference cultivars.

A panel of 28 winter oilseed rape cultivars was grown hydroponically to measure biomass production and root morphological traits (Table 1), in response to the nitrate supply. Mean seed weight at sowing (MSW) showed large genotypic variation and was almost double between Bonanza and DK Expertise cultivars (Supplementary Table 1). Representative root organs of genotypes cultivated with 0.2 mM (N−) or 5.0 mM (N+) nitrate supplies are presented in Figure 1. On average for the diversity panel, the shoot biomass (S, +17.8%) and the total biomass (R+S, +14.5%) increased, the root-to-shoot biomass ratio (R:S, −18.2%) decreased, whereas the root biomass (R) was not different in seedlings treated with N+ compared to those with N−. The length of primary root (L_PR_, +30.5%), the length of primary root zone 4 (L_Z4_, +44.9%), the sum of lateral root lengths (ΣL_LR_, +25.8%), the mean length of lateral roots (ML_LR_, +40.5%), the total root length (TRL, +26.3%), and the specific root length (SRL, +21.8%) were more important, the densities of lateral roots in zone 1 (D_LR_-Z1, −31.7%) and in zone 2 (D_LR_-Z2, −12.7%) were less important, whereas the lengths of primary root zone 2 and 3 (L_Z2_, L_Z3_) and the number of lateral roots (N_LR_) did not change during N− compared to N+ conditions (Figures 1, 2C). All mentioned differences between treatments were significant (*P* < 0.01). Root morphology greatly varied among genotypes. For instance, the percentage differences between the two most extreme genotypes were in the range of 28% (Angus *vs.* ES Mambo) and 58% (ES Navigo *vs.* DK Exentiel) for L_PR_, and of 139% (Cristal *vs.* ES Mambo) and 140% (DK Exclamation *vs.* ES Mambo) for ΣL_LR_, respectively, at N− and N+ (Figures 1, 2A,B). The responsiveness of cultivars to N depletion (i.e., increase/decrease of one trait value in response to N−) was also assessed (Figure 2C). A large variation in phenotypic plasticity was observed, with cultivars poorly or greatly responsive to N supply for L_PR_ (e.g., ES Vito *vs.* Angus) and ΣL_LR_ (e.g., Fernando KWS *vs.* ES Vito).

#### Variance and Multivariate Analyses With Traits Measured in Laboratory Environment

**FIGURE 3:**
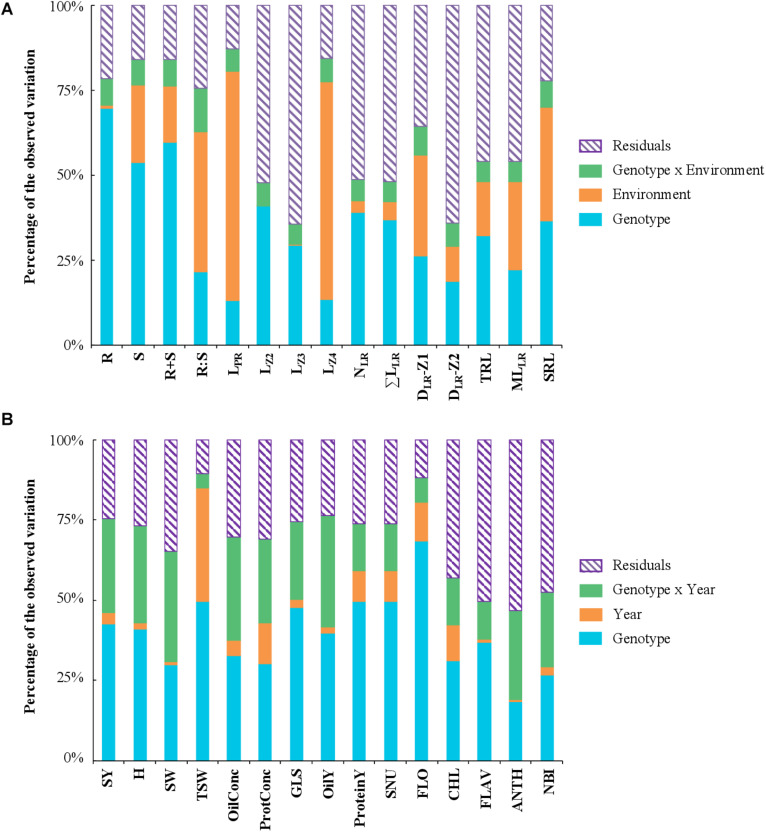
Variance component analysis with phenotypic traits measured in 28 winter oilseed rape cultivars. The histograms show the schematic ANOVA representation for traits measured in hydroponic **(A)** or in field **(B)** conditions. The components of phenotypic variance are **(A)** the genotype/cultivar, environment/nutrition, and interaction (genotype × environment) and residuals as a percentage of the observed variation, and **(B)** genotype/cultivar, year and interaction (genotype × year) and residuals as a percentage of the observed variation. Traits are defined in Tables 1, 2.

A global analysis of variance (ANOVA) assessed the effect of (i) the genotype/cultivar, (ii) the environment/nutrition, (iii) the interaction between the genotype and the environment, and (iv) the residual in the variation of phenotypic traits. The biomass traits (R, S, and R+S) were predominantly influenced by the genotype (54–69% of the total variation), while the R:S ratio by the environment (41%) (Figure 3A). The length of the primary root, and notably L_Z4_, was largely dependent on the environment (67%), while L_Z2_ and L_Z3_ were more reliant on the genotype (38 and 29%, respectively). The remaining traits were also depending on the genotype but generally to a lesser extent. Overall, the interaction (genotype × environment) effect was weak.

**FIGURE 4:**
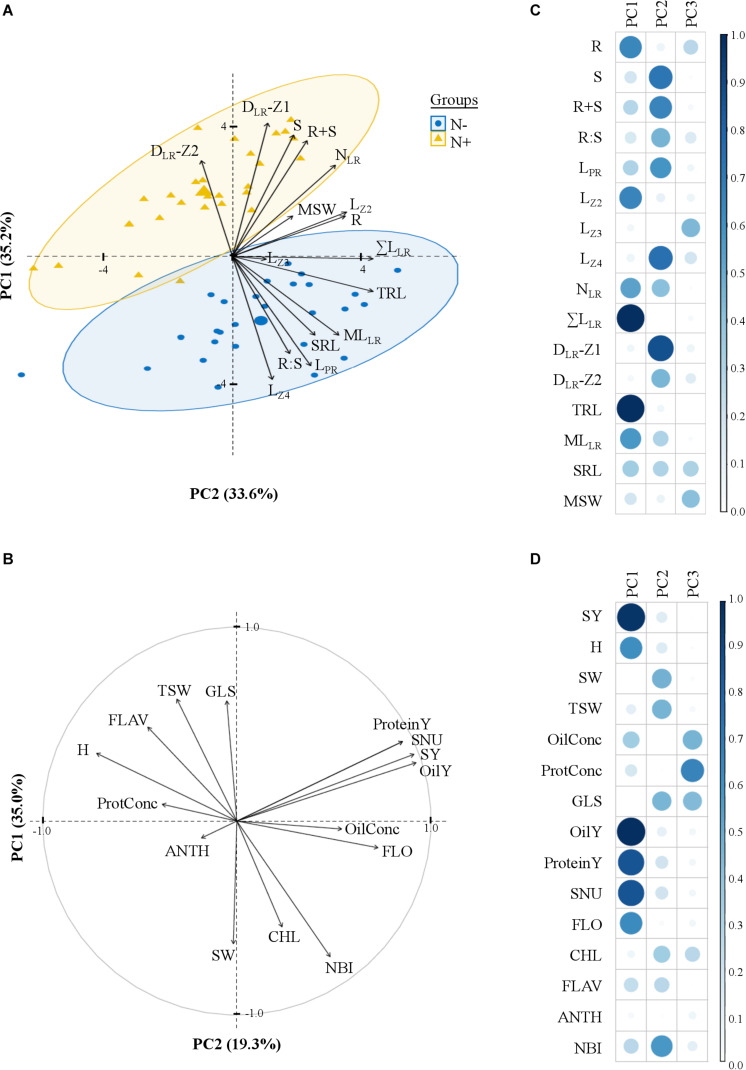
Principal component analysis of traits measured in laboratory and field environments. The biplot graph **(A)** shows 16 variables and 28 cultivars grown in laboratory environment with 5.0 mM (N+) or 0.2 mM (N–) nitrate supplies and the correlation circle **(B)** 15 variables in field environment, with the two first dimensions. Percentages under brackets are those contributed by the first and second principal components. **(C,D)** Representation quality of the variables on the dimensions (values = squared cosines). Traits are defined in Tables 1, 2.

A principal component analysis (PCA) captured the variation in phenotypic traits across the 28 cultivars and the two N treatments (Figures 4A,B). The three first components (PCs), respectively, explained 35.2, 33.6, and 12.9% of the total variation. Some root length traits (TRL, ΣL_LR_, L_Z2_, ML_LR_) and R had mainly loads on PC1 (63%), while other traits (D_LR_-Z1, L_Z4_, S, R+S and L_PR_) on PC2 (61%) and MSW on PC3 (18%).

### In-Field Assays

#### Performance in Field Environment

Field trials were implemented in a complete randomized block design with four replicates, using three control genotypes (DK Exception, DK Expansion, and DK Exstorm) to normalize the data. The field performance of these genotypes during the 4-year trial is given in Table 4. The seed yield and quality trait values were falling in the range of trials conducted for the last 10 years at the CARAH experimental station (min 4.37 t ha^–1^ in 2007, max 6.65 t ha^–1^ in 2015; unpublished data) and of other surveys (Stahl et al., 2017). On average for the panel of cultivars, the seed yield (SY) varied by 38% between the two most contrasting years (2017 and 2019). Smaller variations between the years were observed for the protein (ProtConc) and the oil (OilConc) concentrations in dry seeds (14 and 6%, respectively). Large genotypic variations were found within a range of 140% for the earliness of flowering (FLO) and 25% for the nitrogen balance index (NBI) between the two most contrasting cultivars (Figure 2D).

**TABLE 4:** Seed yield and quality traits of the control cultivars for the four-year trial.

Harvest Year	SY (t ha^–1^)	H (%)	SW (kg hl^–1^)	TSW (g)	ProtConc (%)	OilConc (%)
2016	5.06 ± 0.19	9.1 ± 0.4	65.6 ± 0.5	3.81 ± 0.15	19.7 ± 0.5	47.2 ± 1.1
2017	6.33 ± 0.33	9.8 ± 0.2	66.4 ± 0.3	4.22 ± 0.20	19.2 ± 0.4	48.0 ± 0.5
2018	4.94 ± 0.32	8.1 ± 1.7	64.2 ± 1.2	5.01 ± 0.55	22.1 ± 0.3	45.1 ± 0.4
2019	4.32 ± 0.22	7.7 ± 0.5	64.8 ± 0.2	4.52 ± 0.22	20.9 ± 0.6	45.6 ± 0.3

	**GLS (μmol g^–1^)**	**ProteinY (t ha^–1^)**	**OilY (t ha^–1^)**	**SNU (kg ha^–1^)**		

2016	12.3 ± 0.9	0.91 ± 0.02	2.17 ± 0.13	145 ± 2.5		
2017	10.4 ± 1.6	1.11 ± 0.04	2.76 ± 0.16	177 ± 6.7		
2018	15.5 ± 0.1	1.00 ± 0.07	2.03 ± 0.15	159 ± 11.0		
2019	13.1 ± 1.1	0.82 ± 0.02	1.79 ± 0.14	131 ± 3.3		

*Mean values (4 replicates ± S.D.) for the three control genotypes (DK Exception, DK Expansion, and DK Exstorm). Traits are defined in Table 2.*

#### Variance and Multivariate Analyses With Traits Measured in Field Environment

The data set of the pluriannual field trial was examined with an ANOVA considering the following effects: (i) genotype, (ii) year, (iii) interaction between genotype and year, and (iv) residuals (Figure 3B). The percentage of variation attributed to the genotype varied between 19% for the anthocyanin index (ANTH) and 68% for FLO. The year effect was generally low, except for the thousand seed weight (TSW) for which it accounted for 35%. The genotype × year interaction was overall more important than the year effect and reached 30% for SY. The PCA with field traits revealed three first components explaining, 35, 19.3, and 13% of the total variation respectively. The oil yield (OilY), protein yield (ProteinY), seed nitrogen uptake (SNU), and SY, mainly influenced PC1 (60%) (Figures 4C,D), while PC2 was mostly attributed to the glucosinolate concentration in dry seeds (GLS), the nitrogen balance index (NBI), the specific weight of seeds (SW) and TSW (57%), and PC3 to OilConc and ProtConc at harvest (49%).

### Correlations Between Traits Measured in Laboratory and Field Environments

**FIGURE 5:**
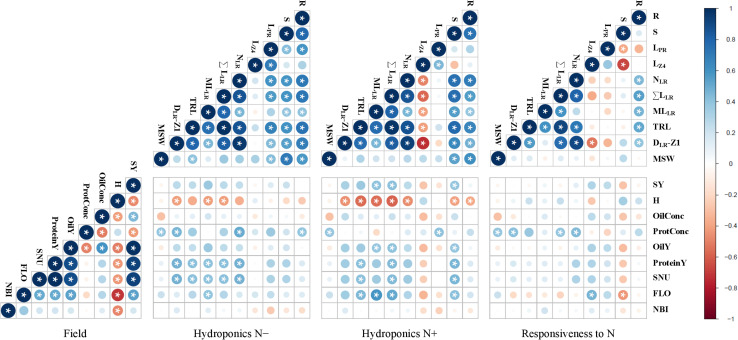
Inter-trait phenotypic correlations in winter oilseed rape genotypes. Biomass production and root morphological traits were measured at 0.2 mM nitrate (N–) or 5 mM nitrate (N+) in hydroponics. Seed yield and seed quality traits as well as optical indices were measured in the field. Circle area and color intensity indicate the strength of the Pearson’s correlation. Blue or red colors indicate positive or negative correlations. Star indicates a correlation coefficient significantly (*P* < 0.05) different from zero. Traits are defined in Tables 1, 2.

Correlations were established firstly between all traits measured in one culture environment, and secondly between all traits measured in laboratory and field environments (Supplementary Table 2). Only traits with important loads on PCs in both environments were considered for drawing the correlograms (Figure 5). Pearson’s correlation matrices were generated considering the two N treatments separately and then the responsiveness to N. In hydroponics, biomass traits (R, S) and root morphological traits associated with lateral roots (N_LR_, ΣL_LR_, ML_LR_, D_LR_-Z1) were positively correlated to each other during both N conditions. These traits were positively correlated with L_PR_ at N−, and negatively correlated with L_Z4_ at N+. The MSW was correlated to R and S in both environments and only to some length parameters (L_PR_, TRL) at N−. In field conditions, FLO was negatively correlated with H. All yield traits were implicitly correlated with SY, while the ProtConc was negatively correlated with OilConc and SY. The nitrogen balance index (NBI), an optical index measured at flowering, was negatively correlated with the seed humidity at harvest (H).

Some root related traits (N_LR_, D_LR_-Z1, R) measured under N− were positively correlated with ProtConc, while ΣL_LR_ and ML_LR_ under N+ were positively correlated with SY. Some other root morphological traits (ΣL_LR_, TRL) measured during both N treatments were positively correlated with the seed N uptake (SNU). The responsiveness to N depletion of some traits related to lateral roots (N_LR_, D_LR_-Z1, ΣL_LR_) was positively correlated with ProtConc, and the responsiveness of S negatively with FLO. No significant (*P* > 0.05) correlation was found between TSW and seedling root traits during both N treatments.

### Genetic Survey of Winter Oilseed Rape Cultivars

**FIGURE 6:**
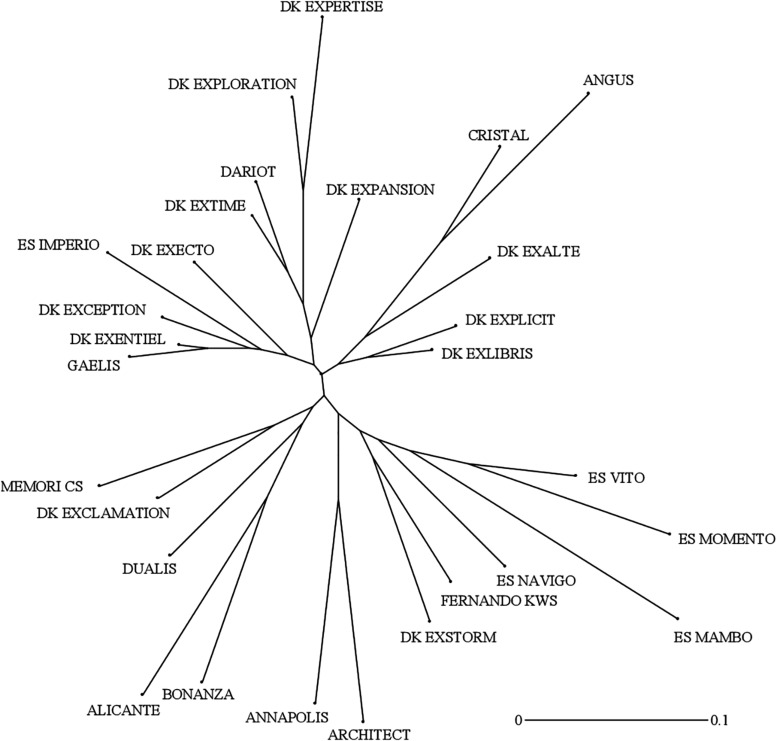
Unweighted Neighbor-Joining (NJ) radial tree showing genetic interrelationships of 28 winter oilseed rape cultivars. The pairwise comparisons were based on dissimilarity coefficients computed from simple matching of allelic data using 17 SSR markers.

The genetic interrelationships among the 28 cultivars were established using 17 polymorphic SSR markers to eventually identify relationships between genetic distances and measured trait distances in laboratory and field environments. A total of 53 alleles were detected in the diversity panel and the number of alleles per marker ranged from 1 to 7, with an average of 3.12 (Table 3). Four markers (BrGMS0667, BrGMS3837, Na12D10 and O112D05) were monomorphic. The allele frequency varied from 1.8% (rare) to 92.9% (common), while the mean was 26.5%. Fifteen out of the 53 total alleles were regarded as rare ones (<5%). The polymorphism information content (PIC) values for all markers ranged from 0.00 to 0.76, with a mean value of 0.29. Only three markers (BrGMS0070, BnGMS0281, and Na12F03) had a PIC value above 0.50. The observed heterozygosity varied from 0.00 to 0.82, with an average of 0.33. The hierarchical clustering using Neighbor-Joining (NJ) generated a radial tree (Figure 6) that set together the most closely related cultivars on common branches, and apart those more genetically distant. When reporting the length of the tree branches between the accession pairs to the scale bar, it appeared that the genetic distances within the panel were overall short.

### Correlations Between Genetic Distance and Phenotypic Trait Distance Matrices

Euclidean distances matrices between cultivars were first calculated for each of the traits measured in hydroponic or field conditions and then, correlated to the genetic dissimilarity matrix computed from the genotyping data, using a Mantel test (Supplementary Table 3). Significant correlations (*P* < 0.05) were observed between genetic dissimilarities and distances among root morphological traits like S (*r* = 0.24), R+S (*r* = 0.19), L_PR_ (*r* = 0.33), L_Z2_ (*r* = 0.33), L_Z4_; (*r* = 0.30), and SRL (*r* = 0.26) measured in N− conditions. Significant correlations were also found between genetic dissimilarities and distances among field traits like SY; (*r* = 0.35), ProtConc (*r* = 0.20), GLS (*r* = 0.17), OilY (*r* = 0.33), SNU (*r* = 0.30), and FLO (*r* = 0.21).

## Discussion

There is a growing awareness among crop breeders concerning research on root being neglected compared to shoot and reproductive organs. Optimizing root morphology is an important strategy for increasing water and nutrient uptake and coping with soil fertility problems (Den Herder et al., 2010; Lynch, 2013). This study explored the genetic diversity of root morphology in a panel of modern winter oilseed rape cultivars, using a laboratory set-up, and was followed with field validation. The results, discussed hereafter, strengthen the premise that root morphological traits could be successful indicators of field performance. Furthermore, phenotypic and marker-trait correlations launch some prospects for crop breeding programs.

### The Root to Shoot Biomass Allocation Is a Key Adaptive Strategy to Nitrogen Availability but Does Not Show Trade-Off Among Cultivars

The root morphological traits of rapeseed are rapidly responding to N availability, with differences reported as early as two days after N depletion (Qin et al., 2019). Nonetheless, seed nutrition may support the growth of seedlings, and attenuate differences between N treatments. This high-throughput hydroponic culture system envisages sequentially a two-day germination step with distilled water (to deplete N storage) and a four-day culture with two divergent N supplies. Hence, the method is suitable for discriminating root organ morphologies at a young development stage (Figure 1). Some traits were more depending on the genotype (e.g., R, N_LR_), while other ones on the N nutrition (e.g., R:S, L_PR_). Several authors share the foundation that a profuse crop root system exploring a large volume of soil would limit N leaching (Lynch, 2013; Li et al., 2016; Thorup-Kristensen and Kirkegaard, 2016). However, conflicting opinions may be expressed regarding to a possible trade-off between large root system size, contributing to N absorption capacity, and the metabolic costs associated with the growth and maintenance of that organ, which can *in fine* have an impact on NUpE. Results showed increased root to shoot biomass ratio during N− conditions, but a positive correlation between root biomass production and root length, and shoot biomass production across the two N treatments (Figure 5). The responsiveness of shoot and root dry biomass production to N deprivation were not correlated. This indicates a positive impact of increased root production on above ground biomass while comparing cultivars. An allometric effect cannot be excluded, where plants with greater biomass having greater root morphological features (Niklas, 2004). The length of primary (especially L_Z4_) and of lateral roots responded distinctly to N availability (Louvieaux et al., 2018; Qin et al., 2019). The inverse relationship between L_Z4_ and other lateral root related traits (N_LR_, ΣL_LR_, D_LR_-Z1, ML_LR_) at N+, point out different strategies deployed to modulate horizontal or vertical expansions of the root system. The N responsiveness of L_PR_ and L_Z4_ (i.e., an increase of L_PR_ and L_Z4_ in response to N−) was negatively correlated with S biomass responsiveness (i.e., a decrease of S biomass in response to N−) (Figure 5). This indicates that cultivars with invariable rooting depth could produce more shoot biomass during N depletion.

### Root Traits Observed at a Seedling Stage Are Predictors of Field Performance

The cultivars were tested in a pluri-annual field trial for determining some NUE and yield components. The N taken up by roots and utilized for producing seeds (SNU) was considered as a proxy for NUE. The data showed the genotype effect was overall the most important and that genotype × year interaction generally greater than the year effect (Figure 3). Indeed, cultivars performed differently from year to year, and this highlights the importance of conducting trials over several seasons. FLO was negatively correlated with H and positively with SY (Figure 5). This implies that early-flowering cultivars achieved seed maturity sooner and performed better than late-flowering ones.

Some traits observed at a young developmental stage in laboratory conditions were significantly correlated with field parameters. The positive correlation between lateral root traits (ΣL_LR_, ML_LR_) measured in hydroponics during N+ and in-field SY (Figure 5), evokes that rapid lateral root development of seedlings is a desirable field characteristic, as stated by Ulas et al. (2012) and Thomas et al. (2016b). Seed germination vigor and rapid radicle growth may enhance seedling survival and ultimately yield (Hatzig et al., 2015; Thomas et al., 2016b; Boter et al., 2019). In this study, the seed weight at sowing had no marked influence neither on seedling root size neither on yield components (Figures 4, 5), in line with Hatzig et al. (2015) but contrary to Thomas et al. (2016a).

Besides, root traits (ΣL_LR_, TRL) measured under both N treatments were positively correlated with SNU, signifying that root phenotypes could be considered for screening NUE components. Measurements of root system morphology and total biomass at harvest (roots, stems, leaves, pods, and seeds) should be considered in future field trials to better evaluate the total N uptake and the reallocation to seed organs, but this is hardly achievable in field conditions. The 4-year trial was marked with a severe rain deficit, in such conditions root traits may also be important for water absorption and maintaining yield under drought conditions (Den Herder et al., 2010; Comas et al., 2013).

The lateral root traits (N_LR_, ΣL_LR_, D_LR_-Z1) were correlated positively with FLO and negatively with H (Figure 5). The seed maturation is marked by H decreased (Elias and Copeland, 2001; Sadeghi et al., 2010). Since harvest was simultaneously done under the same climatic conditions, a low H value reflects an early seed maturity. Presumably, plants with greater root development reach more rapidly seed maturity. Genotypes flowering early better synchronize N mobilization with the pods demand and potentially have an extended seed filling period (Malagoli et al., 2005; Stahl et al., 2016). Therefore, optimizing flowering time is an important breeding target (Schiessl et al., 2014).

Breeding efforts to select modern varieties achieving great oil yield could possibly be the reason for which SY and OilConc traits are intricated (Stahl et al., 2016, 2017). The SY was negatively correlated with ProtConc, but positively with ProteinY (Figure 5). This confirms that yield *per se* is more decisive than seed N concentration with the purpose of improving ProteinY and seed N uptake (SNU). However, ProtConc was positively correlated with root traits measured during N− (N_LR_, D_LR_-Z1, R) and N+ (L_PR_) conditions.

The N responsiveness of ΣL_LR_ (i.e., an increase of this trait in response to N−) was positively correlated with ProtConc (Figure 5), meaning that cultivars with important lateral root growth plasticity have greater protein concentration in seeds. The N responsiveness of S biomass (i.e., a decrease of S in response to N−) and the N responsiveness of L_Z4_ (i.e., an increase of L_Z4_ in response to N−) were negatively and positively correlated with FLO, respectively (Figure 5). This indicates that cultivars with major impact on S biomass and profound impact on L_Z4_ during N depletion were flowering early.

These results corroborate recent reports on oilseed rape, but also on other crops like maize and wheat, in which root traits observed at a seedling stage could predict field performance (Canè et al., 2014; Ali et al., 2015; Thomas et al., 2016b).

### The Genetic Variability of Root Traits Can Be Exploited to Develop Markers for Assisted Breeding to Improve Soil Resource Capture

The assessment of the genetic diversity among the hybrid varieties with SSR markers indicated a rather narrow genetic basis (Table 3 and Figure 6). Four markers were monomorphic, despite filling internal tests with different germplasms (our unpublished data). Besides, the PIC values and the observed heterozygosity were lower than in other reports using the same markers (Li et al., 2013; Chen et al., 2017, 2020). The small number of detected alleles per marker and the high disparities occurring in the allelic frequencies between common and rare alleles, explained together the lower PIC scores in the present study (Table 3). However, the great variability of biomass production and of root morphology within that nested gene pool of modern cultivars, indicates that these traits may constitute an exploitable resource in a breeding effort to ameliorate NUE. Furthermore, the weak relationships between the genetic dissimilarities and phenotypic distances data measured during N− conditions, are encouraging us to extend the study to more genotypes from more diverse origins using denser molecular markers. Mapping approaches in a wider *B. napus* gene pool could be adopted to characterize the allelic variation. Such traits could be incorporated in superior cultivars by genomic and marker-assisted selection strategies.

This report with a small panel of modern winter oilseed rape cultivars compared root phenotypes, field harvest and NUE components. Similar investigation sought to evaluate genotypic variation for root systems and NUE were successfully conducted in core sets of other crop species (Yang et al., 2019; Iqbal et al., 2020). To strengthen this pilot experiment, further studies with a larger diversity panel should assess yield stability in multi-environment trials and across N rates (Thorup-Kristensen and Kirkegaard, 2016; Stahl and Snowdon, 2018). Selection of seedling root traits that could predict field performance, would open a cost-effective way to facilitate introgression of root morphology in rapeseed breeding programs.

## Conclusion

Below-ground phenotyping of the root organ can be tedious in breeding programs. Root traits measured in laboratory culture were to a certain degree predictive of field performance. Such high-throughput screen could be applied to a larger mapping population to identify genes and alleles shaping root morphology, for selection targets in breeding programs to use soil resources more efficiently. This exploratory work is supporting possible genetic improvements for the root morphology of modern oilseed rape cultivars.

## Data Availability Statement

The raw data supporting the conclusions of this article will be made available by the authors, without undue reservation.

## Author Contributions

CH and JL contributed to the conceptualization and funding acquisition. JL contributed to the methodology, field and laboratory investigations, formal analysis, and writing-original draft. MS contributed to the genotyping investigation. CH, MS, and JL contributed to the writing-review and editing. All authors contributed to the article and approved the submitted version.

## Conflict of Interest

The authors declare that the research was conducted in the absence of any commercial or financial relationships that could be construed as a potential conflict of interest.
