# Supplementary material for: Root Morphological Traits of Seedlings Are Predictors of Seed Yield and Quality in Winter Oilseed Rape Hybrid Cultivars
Source: Front Plant Sci. 2020 Oct 15;11:568009. doi: 10.3389/fpls.2020.568009 (PMC7593254; doi:10.3389/fpls.2020.568009)
Supplement: Supplementary file 1 [file Presentation_1.PPTX]

## Slide 1
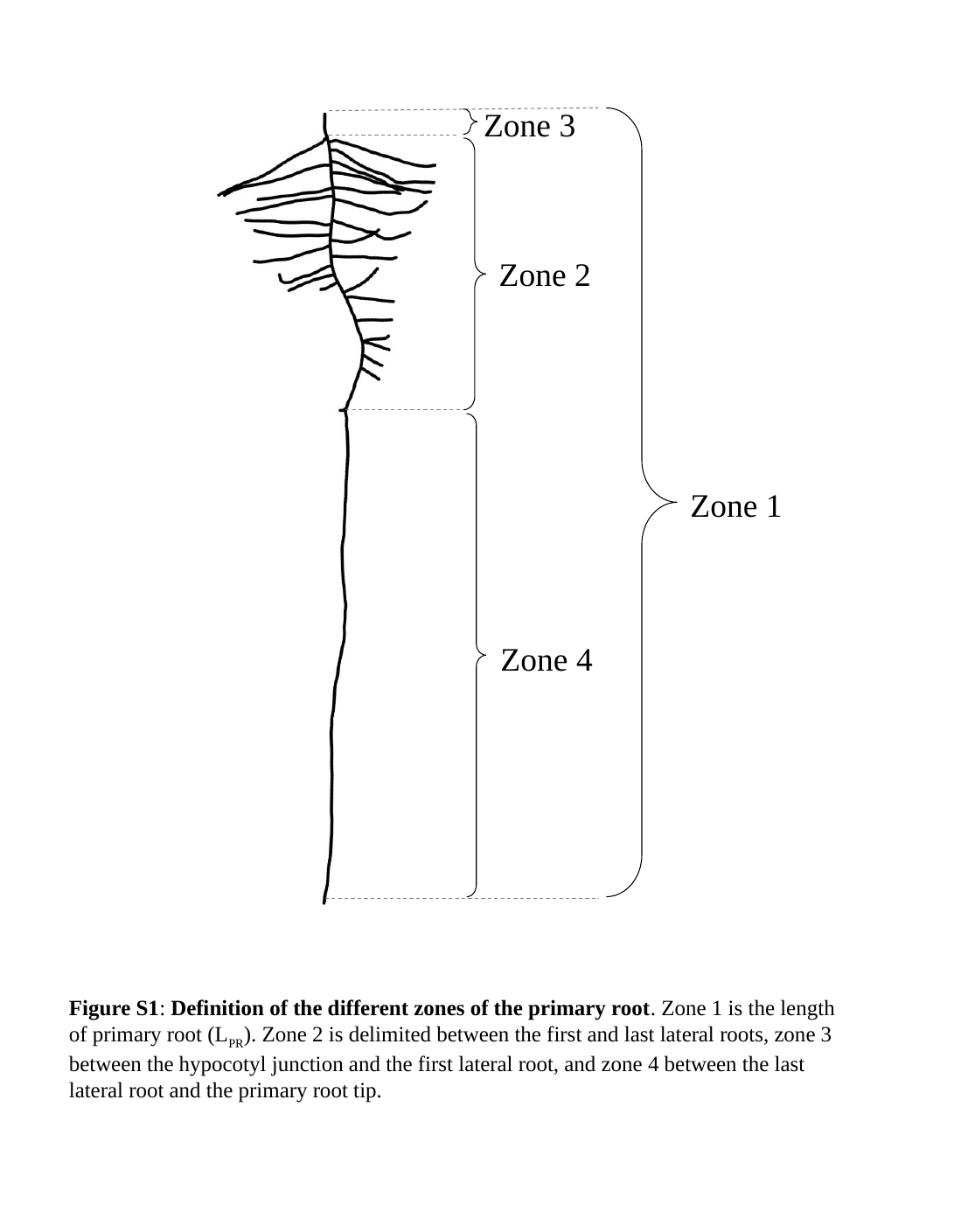

Zone 3
Zone 2
Zone 1
Zone 4
Figure S1: Definition of the different zones of the primary root. Zone 1 is the length of primary root (LPR). Zone 2 is delimited between the first and last lateral roots, zone 3 between the hypocotyl junction and the first lateral root, and zone 4 between the last lateral root and the primary root tip.
