# Supplementary material for: Root Morphological Traits of Seedlings Are Predictors of Seed Yield and Quality in Winter Oilseed Rape Hybrid Cultivars
Source: Front Plant Sci. 2020 Oct 15;11:568009. doi: 10.3389/fpls.2020.568009 (PMC7593254; doi:10.3389/fpls.2020.568009)
Supplement: Supplementary file 2 [file Table_1.DOCX]

**Table S1**: **List of 28 winter oilseed rape cultivars tested and mean seed weight.**

| **Cultivar name** | **Type** | **Registration year** | **Country of registration** | **Field trial years** | **MSW**  **[mg] ± s.d.** |
| --- | --- | --- | --- | --- | --- |
| ALICANTE | HR | 2015 | FR | 2 | 5.12 ± 0.02 |
| ANGUS | HR | 2016 | FR | 2 | 6.08 ± 0.02 |
| ANNAPOLIS | HR | 2016 | FR | 2 | 5.00 ± 0.02 |
| ARCHITECT | HR | 2017 | FR | 3 | 5.95 ± 0.03 |
| BONANZA | HR | 2011 | FR | 2 | 3.16 ± 0.03 |
| CRISTAL | HR | 2015 | FR | 2 | 6.87 ± 0.14 |
| DARIOT | HR | 2015 | FR | 2 | 4.62 ± 0.05 |
| DK EXALTE | HR | 2014 | FR | 2 | 5.51 ± 0.01 |
| DK EXCEPTION* | HR | 2014 | FR | 4 | 4.52 ± 0.04 |
| DK EXCLAMATION | HR | 2016 | FR | 2 | 5.30 ± 0.03 |
| DK EXECTO | HR | 2016 | HU | 2 | 5.19 ± 0.08 |
| DK EXENTIEL | HR | 2014 | FR | 2 | 5.77 ± 0.06 |
| DK EXLIBRIS | HR | 2016 | FR | 3 | 4.82 ± 0.02 |
| DK EXPANSION* | HR | 2015 | FR | 4 | 4.78 ± 0.02 |
| DK EXPERTISE | HR | 2011 | FR | 2 | 7.17 ± 0.02 |
| DK EXPLICIT | HR | 2011 | FR | 3 | 5.18 ± 0.06 |
| DK EXPLORATION | HR | 2014 | FR | 2 | 5.79 ± 0.01 |
| DK EXSTORM* | HR | 2011 | FR | 4 | 5.34 ± 0.02 |
| DK EXTIME | HR | 2016 | UK | 2 | 5.77 ± 0.04 |
| DUALIS | HR | 2016 | FR | 3 | 5.51 ± 0.02 |
| ES IMPERIO | HR | 2015 | FR | 2 | 6.23 ± 0.07 |
| ES MAMBO | L | 2014 | FR | 3 | 4.77 ± 0.03 |
| ES MOMENTO | HR | 2014 | UK | 3 | 5.03 ± 0.07 |
| ES NAVIGO | HR | 2019 | FR | 2 | 5.34 ± 0.07 |
| ES VITO | HR | 2017 | SK | 2 | 5.22 ± 0.07 |
| FERNANDO KWS | HR | 2014 | FR | 2 | 5.62 ± 0.02 |
| GAELIS | HR | 2015 | FR | 2 | 5.03 ± 0.01 |
| MEMORI CS | HR | 2017 | FR | 2 | 5.59 ± 0.02 |
| ** = control varieties in pluriannual field trials.* FR = France; HR = hybrid; HU = Hungary; L = line; MSW = mean seed weight at sowing determined by weighting three subsamples of 500 seeds; SK = Slovakia; UK = United Kingdom. More information available on EU Plant Variety Database (v3.2). | | | | | |
