# Supplementary material for: Root Morphological Traits of Seedlings Are Predictors of Seed Yield and Quality in Winter Oilseed Rape Hybrid Cultivars
Source: Front Plant Sci. 2020 Oct 15;11:568009. doi: 10.3389/fpls.2020.568009 (PMC7593254; doi:10.3389/fpls.2020.568009)
Supplement: Supplementary file 4 [file Table_3.DOCX]

**Table S3: Correlations between dissimilarity matrix obtained with SSR markers and distance matrices for traits measured in laboratory or field environments.** *r* = coefficient correlation between matrices using Mantel test, *P* = p-value. Stars indicate coefficient correlation with *P*<0.05.

| **Traits measured in laboratory environment (N−)** | | | | | | | | | | | | | | | | | |
| --- | --- | --- | --- | --- | --- | --- | --- | --- | --- | --- | --- | --- | --- | --- | --- | --- | --- |
|  | R | S | R+S | R :S | L_PR_ | L_Z2_ | L_Z3_ | L_Z4_ | N_LR_ | ΣL_LR_ | D_LR_-Z1 | D_LR_-Z2 | TRL | ML_LR_ | SRL | |  |
| *r* | 0.148 | 0.239* | 0.186* | -0.101 | 0.332* | 0.274* | -0.103 | 0.301* | 0.177 | 0.103 | 0.172 | 0.141 | 0.153 | 0.053 | 0.255* | |  |
| *P* | 0.094 | 0.015 | 0.049 | 0.831 | 0.001 | 0.010 | 0.772 | 0.001 | 0.090 | 0.194 | 0.088 | 0.115 | 0.114 | 0.307 | 0.014 | |  |
| **Traits measured in laboratory environment (N+)** | | | | | | | | | | | | | | | |  |  |
|  | R | S | R+S | R :S | L_PR_ | L_Z2_ | L_Z3_ | L_Z4_ | N_LR_ | ΣL_LR_ | D_LR_-Z1 | D_LR_-Z2 | TRL | ML_LR_ | SRL | |  |
| *r* | -0.005 | 0.083 | 0.077 | -0.065 | -0.160 | 0.072 | 0.015 | -0.009 | 0.010 | 0.092 | -0.062 | 0.106 | 0.058 | 0.046 | 0.076 | |  |
| *P* | 0.505 | 0.233 | 0.238 | 0.733 | 0.887 | 0.194 | 0.434 | 0.512 | 0.449 | 0.212 | 0.722 | 0.164 | 0.295 | 0.347 | 0.267 | |  |
| **Traits measured in laboratory environment (responsiveness to N−)** | | | | | | | | | | | | | | | |  |  |
|  | R | S | R+S | R :S | L_PR_ | L_Z2_ | L_Z3_ | L_Z4_ | N_LR_ | ΣL_LR_ | D_LR_-Z1 | D_LR_-Z2 | TRL | ML_LR_ | SRL | |  |
| *r* | -0.038 | 0.060 | 0.102 | -0.077 | -0.146 | 0.072 | -0.050 | 0.075 | 0.050 | -0.079 | 0.049 | 0.147 | -0.052 | -0.088 | -0.085 | |  |
| *P* | 0.632 | 0.275 | 0.182 | 0.722 | 0.842 | 0.288 | 0.659 | 0.202 | 0.340 | 0.770 | 0.339 | 0.139 | 0.720 | 0.788 | 0.811 | |  |
| **Traits measured in field environment** | | | | | | | | | | | | | | | |  |  |
|  | SY | H | SW | TSW | OilConc | ProtConc | GLS | OilY | ProteinY | SNU | FLO | CHL | FLAV | ANTH | NBI | |  |
| *r* | 0.350* | 0.152 | -0.033 | -0.007 | 0.074 | 0.200* | 0.173* | 0.329* | 0.301* | 0.301* | 0.212* | -0.199 | -0.007 | 0.083 | -0.005 | |  |
| *P* | 0.003 | 0.143 | 0.640 | 0.516 | 0.179 | 0.043 | 0.023 | 0.007 | 0.007 | 0.008 | 0.044 | 0.970 | 0.511 | 0.301 | 0.499 | |  |
